# Intravascular Lithotripsy and Aortic Bare-Metal Stenting: A Low-Profile Solution for the Treatment of Heavily Calcified Aorto-Iliac Disease

**DOI:** 10.1177/15266028241270650

**Published:** 2024-08-16

**Authors:** Nikolaos Konstantinou, Nikolaos Tsilimparis, Konstantinos Stavroulakis

**Affiliations:** 1 Department of Vascular Surgery, University Hospital, LMU Munich, Munich, Germany

**Keywords:** intravascular lithotripsy, bare-metal stent, low-profile, aorto-iliac occlusive disease, peripheral arterial disease

## Abstract

**Purpose:**

To present a novel technique for the treatment of heavily calcified aorto-iliac disease using intravascular lithotripsy (IVL) and self-expanding bare-metal stents (BMS).

**Technique:**

We present our experience with 4 cases of calcified aorto-iliac disease that were treated with IVL as vessel preparation followed by BMS deployment. Intravascular lithotripsy was performed using a 7-mm or 8-mm Shockwave catheter from 1 access and a non-compliant balloon introduced from the second access in a “hugging-balloon” configuration. Afterward, a self-expandable BMS is deployed in the infrarenal aorta and additional bare-metal balloon-mounted stents are deployed in the iliac arteries as needed. This technique provides a low-profile solution with only 6- and 7-French introducers, preservation of the collateral circulation while also preserving the option for an up-and-over approach in the future. Technical success was achieved in all cases and no periprocedural complications were observed.

**Conclusion:**

Intravascular lithotripsy in combination with BMS for the infrarenal aorta and the aortic bifurcation seems to be a safe and effective low-profile treatment option for heavily calcified lesions. Large-scale studies with long-term follow-up are needed to validate our positive early results.

**Clinical Impact:**

Endovascular treatment of heavily calcified aortoiliac disease poses significant challenges, including the risk of rupture and dissection. The proposed technique uses intravascular lithotripsy and bare-metal stenting of the aortic bifurcation and represents a low-profile solution that preserves collaterals and potentially reduces the risk of dissection with IVL vessel preparation.

## Introduction

Surgical treatment of aorto-iliac occlusive disease (AIOD) has been traditionally considered the gold standard approach; however, it is associated with prolonged hospital stay and increased periprocedural morbidity and mortality.^1,2^ Endovascular therapy has emerged in this setting as a minimally invasive alternative to surgery with less complications and acceptable long-term patency rates, leading to an “endo-first” approach in most cases.^1,3^

One of the biggest challenges in the endovascular treatment of AIOD, however, arises in severely calcified lesions,^4^ in which the risk for major complications including aortic rupture, dissection, and distal embolization significantly increases.^5,6^ The covered endovascular repair of the aortic bifurcation (CERAB) technique^7^ was proposed as a means to mitigate these complications. However, the larger profile of the introducers of large-lumen stent grafts that have to be navigated through often calcified iliac vessels remains a significant limitation of this technique. Additionally, the aggressive balloon expansion needed for the placement of the stent grafts might increase the risk of rupture in case of coral-reef aortic disease, while severe calcification might lead to an under-expansion of the deployed stents.

To address these issues, the use of intravascular lithotripsy (IVL) for heavily calcified aortic lesions has been proposed, either as a stand-alone treatment or combined with plain balloon angioplasty,^8,9^ showing some promising early results. We present herein our experience using a novel technique combining IVL and low-profile, large-lumen, self-expanding bare-metal stents (BMS) for the treatment of these heavily calcified aortic lesions.

## Technique Description

A high-quality computed tomography (CT) angiography with a slice thickness of 1 mm or less is acquired from every patient for preoperative planning. Written informed consent was taken from every patient the day before the procedure, informing them also about the off-label nature of device use in aortic lesions. The procedure is performed in a hybrid theater under local anesthesia. A percutaneous 7- or 6-French femoral access is used in every case with additional brachial access if deemed necessary according to the lesion characteristics in the preoperative planning. The lesion is then navigated using an Advantage™ hybrid wire with hydrophilic tip (Terumo Medical, Tokyo, Japan) followed by an aortography to ensure intraluminal guidewire placement. After bilateral crossing, two 0.014-inch guidewires are positioned in the aorta central to the target lesion. An intravascular ultrasound (IVUS) is then performed using a Reconnaissance™ 0.018-inch over-the-wire catheter (Philips NV, Amsterdam, Netherlands) to assess lesion characteristics and plan stent deployment. Afterward, the lesion is dilated with an under-dimensioned non-compliant balloon with about half the diameter of the selected IVL catheter (in most cases 4–5 mm) to facilitate the safe passage of the IVL catheter through the calcified aortic lesion and ensure sufficient circumferential contact with the calcified plaques.

Intravascular lithotripsy is then performed using a 7-mm M5+ or 8-mm M5+ Shockwave™ catheter (Shockwave Medical, Santa Clara, CA) introduced from one access vessel and a 6- or 8-mm (according to the diameter of the infrarenal aorta) non-compliant plain old balloon angioplasty (POBA) catheter is introduced from the second access vessel and placed in a “hugging-balloon” configuration. The IVL catheter is placed with the middle emitter at approximately the center of the lesion and is inflated at 4 atm (subnominal), whereas the POBA catheter is inflated at approximately 6 atm (subnominal) to achieve a combined diameter of about 12–14 mm. Lithotripsy is then initiated with 3 cycles of 30 pulses each, with the sound waves being emitted from both balloons through the hugging-balloon configuration. The IVL catheter is repositioned as needed to cover the entire aortic lesion between each cycle. The IVL and POBA catheter are then swapped places, with the IVL catheter being introduced through the contralateral access and the plain balloon through the first one to achieve homogenous sound wave distribution around the entire vessel wall. The balloons are inflated as previously described and an additional lithotripsy procedure with 3 cycles of 30 pulses each is performed. A kissing IVL technique is not performed. The remaining pulses are then delivered to treat the target lesions based on the findings of the angiogram and the IVUS imaging with the aim to reduce the stenosis of the target lesion to less than 30%.

Afterward, a bare-metal, large-lumen, self-expanding stent is deployed in the infrarenal aorta, usually an E-Luminexx vascular stent (BD, Franklin Lakes, NJ), which is dilated using a 12- or 14-mm non-compliant balloon. The self-expanding stent can be deployed at the level of the aortic bifurcation. To account for the distal “stent-jump” during stent deployment, the distal stent part is placed at the level of the origin of the contralateral common iliac artery. Sizing of the stent is determined from the preoperative CT scan as well as IVUS after the IVL treatment; we usually aim for minimal oversizing up to a maximum of 10% more than the diameter of the perfused lumen. Angiography and IVUS are used to control the results. The procedure is completed with IVL and iliac stenting with balloon-mounted stents as necessary, according to the preoperative planning and angiography findings. The iliac stents can be deployed shortly over the aortic bifurcation without compromising a future up-and-over approach for the treatment of lower limb atherosclerosis. Closure devices are then used for the femoral access site (Angioseal™ from Terumo or Proglide™ from Abbott Cardiovascular, Chicago, IL), while manual compression for the brachial access is employed.

## Case Reports

From May 2022 to July 2023, 4 patients underwent IVL with bare-metal stenting for calcified aorto-iliac lesions in a single tertiary center. Each case is discussed in detail below, and an overview of the clinical characteristics of each patient can be found in Table 1. Pre- and post-treatment imaging can be found in Supplemental materials.

**Table 1.** Demographic and Clinical Characteristics of the Presented Cases.

Case number	Demographics	Rutherford classification	Clinical presentation	Aortic diameter (mm)	Extension of pathology above aortic bifurcation (mm)	Procedure	Discharge	Follow-up
1	78-yo female	6	Gangrenous left small toe, rest pain right leg; *Staphylococcus aureus* bacteremia	13.6	35	1. 7-mm IVL with kissing balloons and stenting infrarenal aorta (Luminex 14 × 40), bare-metal stenting right external iliac, minor amputation left small toe 2. Revascularization of the left SFA with DCB-angioplasty and spot-stenting	POD 25	Alive, no major amputation, no claudication
2	66-yo male	3	Intermittent claudication both legs	13.1	52	7-mm IVL with kissing balloons and BMS infrarenal aorta	POD 3	Alive, no claudication, no major amputation
3	61-yo male	5	Intermittent rest pain, ulcer right second toe	12	17	8-mm IVL with kissing-balloons and BMS infrarenal aorta, kissing-stents common iliac arteries, BMS right external iliac	POD 4	Alive, minor amputation second toe right side on POM 1, no major amputation
4	61-yo male	3	Intermittent claudication both legs	12.7	27	8-mm IVL with kissing-balloons and BMS infrarenal aorta, kissing-stents common iliac arteries	POD 5	Alive, no claudication, no major amputation

## Case 1

A 78-year-old female patient presented with a Rutherford class 6 ischemia of the left foot with gangrene of the fifth toe and concomitant bacteremia with *Staphylococcus aureus* and Rutherford class 4 ischemia of the right leg with rest pain. The patient had undergone endarterectomy of the left common femoral artery (CFA) and stenting of the left common and external iliac arteries one year prior. The past medical history of the patient showed significant comorbidity obstructive pulmonary disease (COPD), coronary artery disease with LAD (left anterior descending artery)-stenting the previous week, type 2 diabetes, pulmonary sarcoidosis, chronic gastritis with previous upper-gastrointestinal tract bleeding, and beginning liver cirrhosis due to non-alcoholic steatohepatitis.

The CT angiography revealed significant calcified stenosis of the infrarenal aorta and right external iliac artery as well as multiple calcified near-total occlusions of both superficial femoral arteries (SFA). The old stents in the left iliac arteries were patent. The patient was deemed to be at a high risk for open surgery, so an endovascular approach was planned.

Bilateral femoral access was established, and an angiography was performed, confirming the CT findings. IVL was performed in the infrarenal aorta with an M5 Shockwave catheter (7 mm) and a 6 × 40 mm Mustang balloon (Boston Scientific, Marlborough, MA) in hugging-balloon configuration, as described above. A 14 × 40 mm E-Luminexx self-expandable stent was then deployed in the infrarenal aorta and was dilated with a 14 × 40 mm non-compliant balloon (Atlas™, BD). Afterward, the iliac arteries on both sides were also treated with IVL due to significant calcification, the stenosis of the right external iliac artery was treated with an 8 × 80 mm Everflex™ self-expandable BMS (Medtronic, Dublin, Ireland). The final angiography showed no residual stenosis. Finally, a transmetatarsal amputation of the small toe was performed and the wound was left open for secondary wound healing due to the infection.

In the following days, the amputation wound was healing only very slowly, so a revascularization of the left superficial artery was decided that occurred on postoperative day (POD) 22. Stenting of the left SFA with an Innova™ 6 × 40 mm self-expandable BMS (Boston Scientific) and dilatation with 2 drug-coated balloons (Ranger™, Boston Scientific) through an up-and-over access from the right side was performed, followed by repeat resection of the distal metatarsal bone of the left small toe. The patient was discharged on POD 25 at home in good clinical condition with improved wound healing.

## Case 2

A 66-year-old male patient, smoker with type-2 diabetes and hypertension, was referred to us due to a Rutherford class 3 ischemia of both legs with intermittent claudication after 100 m of walking. The CT scan revealed a coral-reef infrarenal aorta and an endovascular treatment was planned.

**Figure 1.**
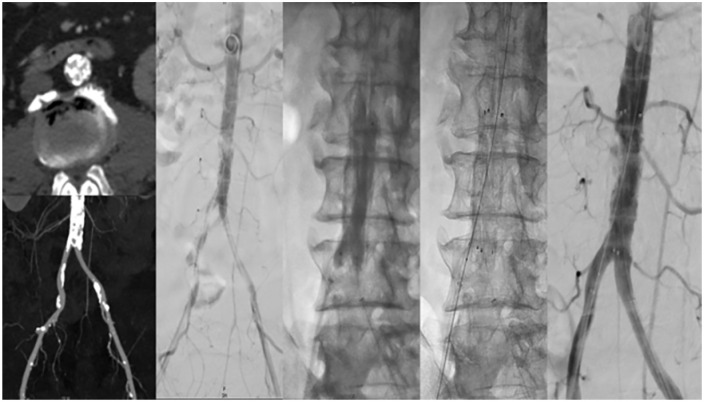
Intravascular lithotripsy and self-expanding bare-metal stent deployment for heavily calcified infrarenal disease.

Femoral access was established on both sides and, after wire passage of the lesion from both sides, IVL with an M5+ 8-mm Shockwave catheter and an 8-mm Mustang balloon in hugging-balloon configuration was performed. A 14 × 50 mm E-Luminexx stent was then deployed in the infrarenal aorta that was dilated with a 12 × 40 mm PowerFlex Pro non-compliant balloon (Cordis, Miami Lakes, FL). The final angiography showed a good result with no residual stenosis (Figure 1).

The patient was discharged home on POD 3 in good clinical condition with no claudication symptoms. The patient was alive and asymptomatic at the 6-month follow-up. No re-intervention was performed.

## Case 3

A 61-year-old male patient was referred to us with Rutherford class 5 ischemia of the right foot with an ulcer of the second toe and rest pain of the left leg. The patient had previously undergone endarterectomy with patchplasty of the CFA on both sides and stenting of the common and external iliac arteries on both arteries in another hospital. Moreover, squamous cell oropharyngeal carcinoma had been diagnosed for the first time a month prior.

The CT scan revealed a coral-reef infrarenal aorta with calcified high-grade stenoses of both common iliac arteries as well as the right external iliac artery. Additionally, significant apposition thrombus in the right CFA with occlusion of both SFAs was detected. An endovascular treatment was planned.

**Figure 2.**
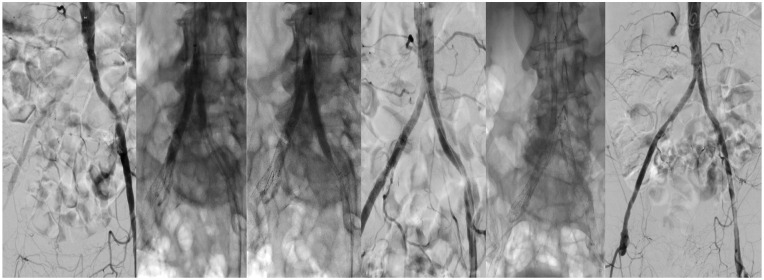
Transfemoral and transbrachial recanalization of the distal aorta and the aortic bifurcation, vessel preparation with IVL, and bare-metal endovascular reconstruction of the aortic bifurcation (BERAB).

Arterial access was established through surgical cut-down of the right brachial artery and puncture of the left CFA. A brachial access was preferred in this case to avoid puncturing inside the thrombus in the right CFA. After wire passage of the lesion from both sides, IVL with an M5+ 8-mm Shockwave catheter and an 8-mm Mustang balloon in hugging-balloon configuration was performed. A 14 × 50 mm E-Luminexx stent was then deployed in the infrarenal aorta that was dilated with a non-compliant balloon. Afterward, 2 Omnilink Elite™ 8 × 37 mm balloon expandable BMS (Abbott Cardiovascular, Chicago, IL) were deployed in the proximal common iliac arteries, one on each side. Finally, the external iliac stenosis on the right side was treated with an 8 × 60 mm Everflex self-expandable BMS. The final angiography showed a good result with no residual stenosis (Figure 2).

The patient was discharged home on POD 4 in good clinical condition. The second toe was amputated 1 month after the treatment in another hospital, and the patient was alive and with no open wounds or ulcers at the 6-month follow-up.

## Case 4

A 61-year-old male patient presented in our outpatient clinic due to a Rutherford class 3 ischemia of both legs with intermittent claudication of the buttocks and upper legs after approximately 100 m of walking. The CT scan revealed a coral-reef infrarenal aorta with heavily calcified proximal common iliac arteries on both sides, as well as a chronic total occlusion of the right SFA.

Bilateral femoral access was established and, after wire passage of the aorto-iliac lesions, IVL with an M5+ 8-mm Shockwave catheter and a 6 × 40 mm Mustang balloon in hugging-balloon configuration was performed. The aortic lesion was then treated with a 14 × 40 mm E-Luminexx stent that was dilated with a non-compliant balloon. The iliac lesions were treated with 2 Dynetic™ 10 × 38 mm balloon expandable BMS (Biotronik, Berlin, Germany) placed in the proximal common iliac arteries, 1 in each vessel. The final angiography showed no residual stenosis. The patient was discharged home on POD 5 in good clinical condition and symptom-free. The patient remained without symptoms 1 month after treatment.

## Discussion

Endovascular treatment of heavily calcified aorto-iliac lesions still poses a number of challenges, including the risk of rupture, flow-limiting dissections, and insufficient luminal gain. The CERAB technique has been proposed to overcome these limitations, but large-lumen stent grafts also require relatively large introducers, that can be difficult to maneuver through calcified iliac vessels and they can also be quite costly. Furthermore, given that the shoulder of the balloon of the stent grafts has a minimum length of 15 mm, the iliac stents have to be expanded up into the distal aorta. This might compromise future interventions of the lower limbs and especially an up-and-over approach. IVL has become an established technique in the treatment of heavily calcified femoropopliteal lesions and is characterized by very low periprocedural complication rates, especially flow-limiting dissections, and distal embolization.^10,11^ The use of IVL has only recently been extended in the iliac segment with promising results, either for the treatment of peripheral artery disease symptoms or to facilitate access for large-bore procedures.^12,13^ The use of IVL in aortic lesions is currently still off-label as the device is not officially approved for use in this vasculature and data on it has been limited only to single case reports.

**Figure 3.**
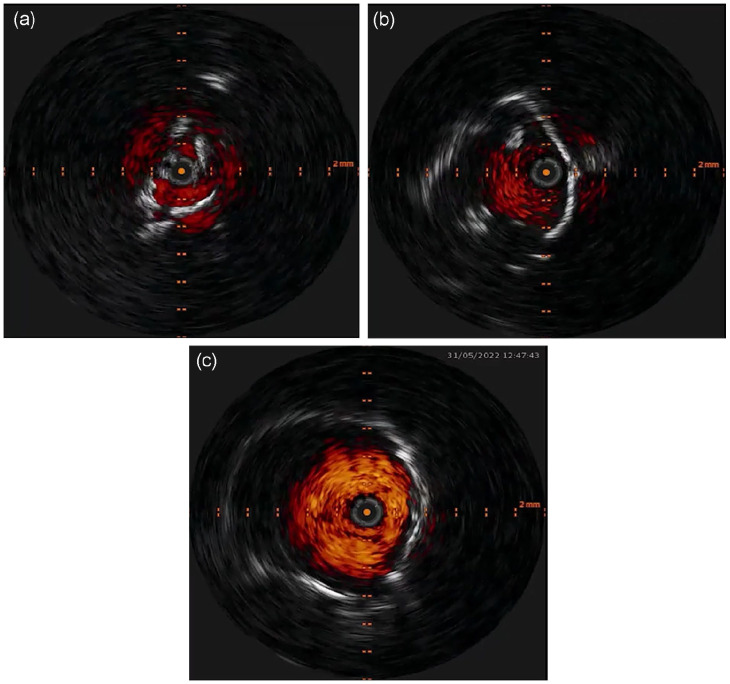
Periprocedural IVUS imaging. (A) baseline IVUS. (B) IVUS post IVL. (C) Final result after bare-metal stent deployment.

In our experience with aorto-iliac IVL we observed significant luminal gains in the aorta and iliac arteries, which has led some to only use IVL angioplasty and nothing else for the treatment of these lesions.^14^ To reach the whole circumference of the aortic wall we used the “hugging-balloon” technique proposed by Chag and Thakre,^8^ which has the added benefit of using only one pulse generator, further reducing operational costs. Although no intravascular imaging was used in this initial report of the technique, limiting the certainty of technique effectiveness, we observed large luminal gains with no flow-limiting dissections using IVUS, which has also been the experience of other authors too^9^ (Figure 3). The aortic stent is dilated with a 12- or 14-mm balloon to achieve optimal stent expansion and usually only low-pressure ballooning is needed after modifying the vessel wall compliance with IVL. Balloon expansion of the stent grafts in CERAB procedures, on the contrary, is usually aggressive dilatation that aims to “break” the calcium, which is also more traumatic for the aortic wall.

**Figure 4.**
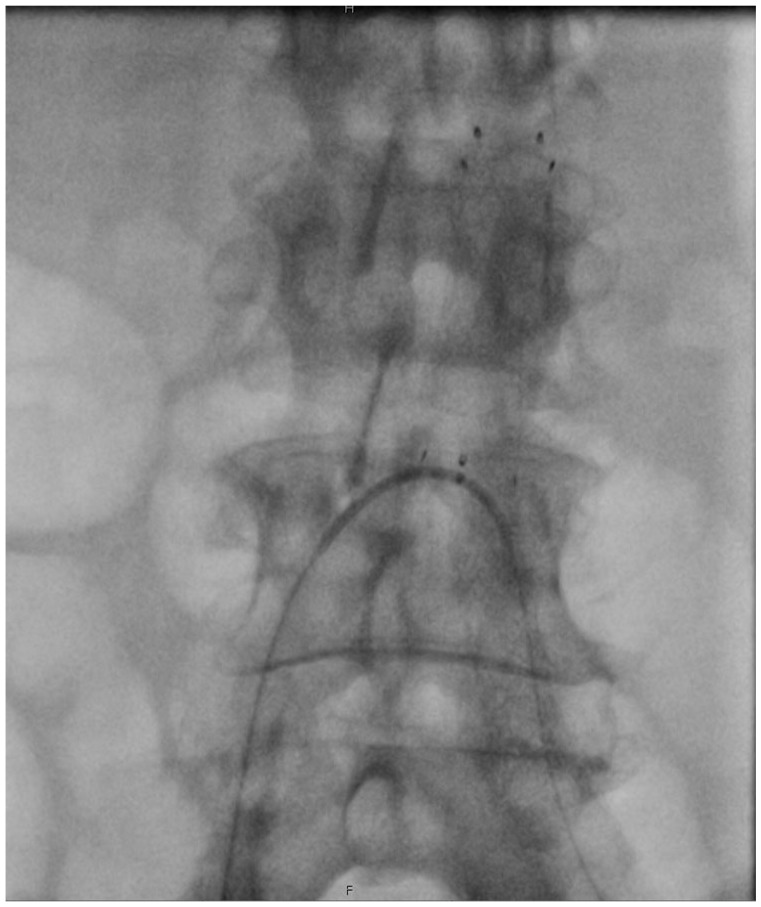
Secondary up and over approach following IVL and BERAB.

The use of self-expanding BMS for aortic stenotic disease is a viable treatment option.^15^ Nonetheless, the use of adjunctive procedures to allow for optimal stent expansion and luminal gain has not been previously described. In our cases, we use aortic stenting after IVL treatment as a means of anti-restenotic treatment to avoid secondary interventions. While there are no available data for vessel preparation prior to stenting of the infrarenal aorta, our results showed a good wall apposition of the aortic stent with no periprocedural complications. We used a low-profile, self-expandable bare-metal stent after aortic IVL, which facilitated an aorto-iliac repair with only 7-French introducers and by preserving both important collateral vessels and the option for future up-and-over interventions (Figure 4). Moreover, using smaller sheaths could also help negotiate occluded or highly calcified iliac vessels more easily and the 7-mm M5+ Shockwave catheter could even be used over a 6F introducer, allowing for an ambulatory treatment of heavily calcified disease. Of note, no arterial ruptures and distal arterial embolization were observed in this high-risk group of patients. However, as this is only an early experience with 4 cases, the generalizability of the results is limited, and a much larger cohort is needed to draw definitive conclusions. Moreover, the cost of IVUS and IVL catheters should be kept in mind when planning these procedures, as these devices are not well or not at all reimbursed in many countries. This effect is, however, offset by the much lower cost of BMS instead of multiple stent grafts.

The introduction of a larger, new-generation IVL catheter (L6) might further simplify the treatment of calcified aortic disease. The catheter will be compatible with a 0.018-inch guidewire and will be available in sizes of up to 12 mm. It is, however, uncertain if a single access treatment of calcified aortic lesions is technically feasible since, to achieve optimal results, some oversizing of the IVL catheter to the calcified vessel is required. That would mean that for the treatment with a single 12-mm L6 catheter, an aortic diameter of 11 mm is necessary, which is quite rare. Moreover, the use of a single 12-mm IVL catheter would lead to excessive oversizing for the iliac arteries when treating aorto-iliac disease.

## Conclusion

Intravascular lithotripsy and BMS of the aortic bifurcation seem to be a safe and effective low-profile treatment option for heavily calcified aorto-iliac lesions. Large-scale studies with long-term follow-up are needed to validate our positive early results.

## Supplemental Material

sj-jpg-6-jet-10.1177_15266028241270650 – Supplemental material for Intravascular Lithotripsy and Aortic Bare-Metal Stenting: A Low-Profile Solution for the Treatment of Heavily Calcified Aorto-Iliac Disease Supplemental material, sj-jpg-6-jet-10.1177_15266028241270650 for Intravascular Lithotripsy and Aortic Bare-Metal Stenting: A Low-Profile Solution for the Treatment of Heavily Calcified Aorto-Iliac Disease by Nikolaos Konstantinou, Nikolaos Tsilimparis and Konstantinos Stavroulakis in Journal of Endovascular Therapy

sj-png-1-jet-10.1177_15266028241270650 – Supplemental material for Intravascular Lithotripsy and Aortic Bare-Metal Stenting: A Low-Profile Solution for the Treatment of Heavily Calcified Aorto-Iliac Disease Supplemental material, sj-png-1-jet-10.1177_15266028241270650 for Intravascular Lithotripsy and Aortic Bare-Metal Stenting: A Low-Profile Solution for the Treatment of Heavily Calcified Aorto-Iliac Disease by Nikolaos Konstantinou, Nikolaos Tsilimparis and Konstantinos Stavroulakis in Journal of Endovascular Therapy

sj-png-2-jet-10.1177_15266028241270650 – Supplemental material for Intravascular Lithotripsy and Aortic Bare-Metal Stenting: A Low-Profile Solution for the Treatment of Heavily Calcified Aorto-Iliac Disease Supplemental material, sj-png-2-jet-10.1177_15266028241270650 for Intravascular Lithotripsy and Aortic Bare-Metal Stenting: A Low-Profile Solution for the Treatment of Heavily Calcified Aorto-Iliac Disease by Nikolaos Konstantinou, Nikolaos Tsilimparis and Konstantinos Stavroulakis in Journal of Endovascular Therapy

sj-png-3-jet-10.1177_15266028241270650 – Supplemental material for Intravascular Lithotripsy and Aortic Bare-Metal Stenting: A Low-Profile Solution for the Treatment of Heavily Calcified Aorto-Iliac Disease Supplemental material, sj-png-3-jet-10.1177_15266028241270650 for Intravascular Lithotripsy and Aortic Bare-Metal Stenting: A Low-Profile Solution for the Treatment of Heavily Calcified Aorto-Iliac Disease by Nikolaos Konstantinou, Nikolaos Tsilimparis and Konstantinos Stavroulakis in Journal of Endovascular Therapy

sj-png-4-jet-10.1177_15266028241270650 – Supplemental material for Intravascular Lithotripsy and Aortic Bare-Metal Stenting: A Low-Profile Solution for the Treatment of Heavily Calcified Aorto-Iliac Disease Supplemental material, sj-png-4-jet-10.1177_15266028241270650 for Intravascular Lithotripsy and Aortic Bare-Metal Stenting: A Low-Profile Solution for the Treatment of Heavily Calcified Aorto-Iliac Disease by Nikolaos Konstantinou, Nikolaos Tsilimparis and Konstantinos Stavroulakis in Journal of Endovascular Therapy

sj-png-5-jet-10.1177_15266028241270650 – Supplemental material for Intravascular Lithotripsy and Aortic Bare-Metal Stenting: A Low-Profile Solution for the Treatment of Heavily Calcified Aorto-Iliac Disease Supplemental material, sj-png-5-jet-10.1177_15266028241270650 for Intravascular Lithotripsy and Aortic Bare-Metal Stenting: A Low-Profile Solution for the Treatment of Heavily Calcified Aorto-Iliac Disease by Nikolaos Konstantinou, Nikolaos Tsilimparis and Konstantinos Stavroulakis in Journal of Endovascular Therapy

sj-png-7-jet-10.1177_15266028241270650 – Supplemental material for Intravascular Lithotripsy and Aortic Bare-Metal Stenting: A Low-Profile Solution for the Treatment of Heavily Calcified Aorto-Iliac Disease Supplemental material, sj-png-7-jet-10.1177_15266028241270650 for Intravascular Lithotripsy and Aortic Bare-Metal Stenting: A Low-Profile Solution for the Treatment of Heavily Calcified Aorto-Iliac Disease by Nikolaos Konstantinou, Nikolaos Tsilimparis and Konstantinos Stavroulakis in Journal of Endovascular Therapy

sj-png-8-jet-10.1177_15266028241270650 – Supplemental material for Intravascular Lithotripsy and Aortic Bare-Metal Stenting: A Low-Profile Solution for the Treatment of Heavily Calcified Aorto-Iliac Disease Supplemental material, sj-png-8-jet-10.1177_15266028241270650 for Intravascular Lithotripsy and Aortic Bare-Metal Stenting: A Low-Profile Solution for the Treatment of Heavily Calcified Aorto-Iliac Disease by Nikolaos Konstantinou, Nikolaos Tsilimparis and Konstantinos Stavroulakis in Journal of Endovascular Therapy
