# Supplementary figures and images for: Intravascular Lithotripsy and Aortic Bare-Metal Stenting: A Low-Profile Solution for the Treatment of Heavily Calcified Aorto-Iliac Disease
Source: J Endovasc Ther. 2024 Aug 16;33(1):113–20. doi: 10.1177/15266028241270650 (PMC12804417; doi:10.1177/15266028241270650)

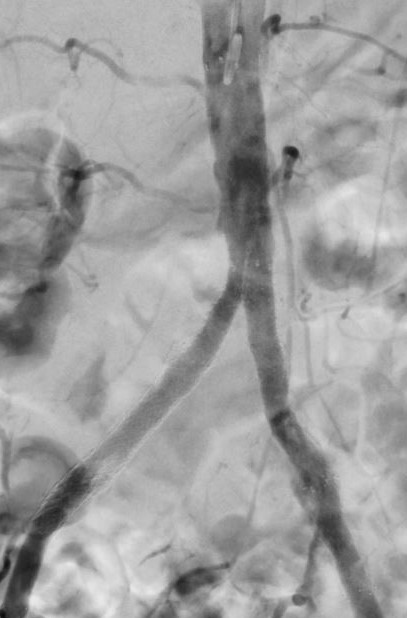

Supplement: sj-jpg-6-jet-10.1177_15266028241270650 – Supplemental material for Intravascular Lithotripsy and Aortic Bare-Metal Stenting: A Low-Profile Solution for the Treatment of Heavily Calcified Aorto-Iliac Disease [file sj-jpg-6-jet-10.1177_15266028241270650.jpg]

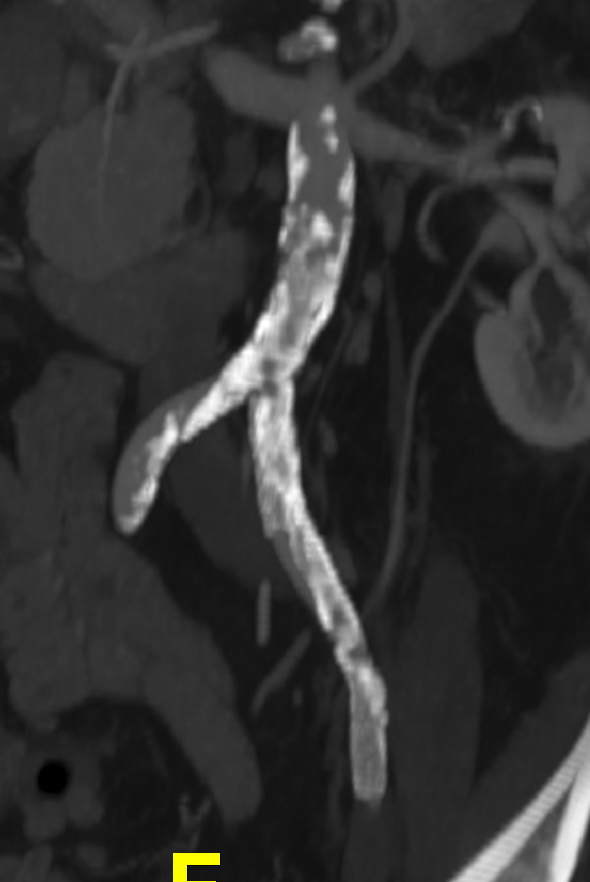

Supplement: sj-png-1-jet-10.1177_15266028241270650 – Supplemental material for Intravascular Lithotripsy and Aortic Bare-Metal Stenting: A Low-Profile Solution for the Treatment of Heavily Calcified Aorto-Iliac Disease [file sj-png-1-jet-10.1177_15266028241270650.png]

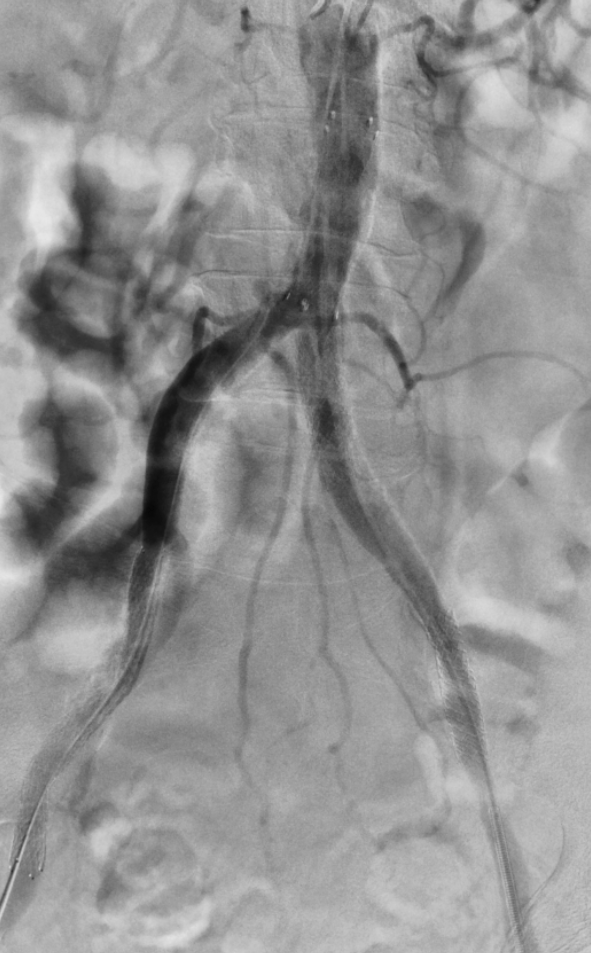

Supplement: sj-png-2-jet-10.1177_15266028241270650 – Supplemental material for Intravascular Lithotripsy and Aortic Bare-Metal Stenting: A Low-Profile Solution for the Treatment of Heavily Calcified Aorto-Iliac Disease [file sj-png-2-jet-10.1177_15266028241270650.png]

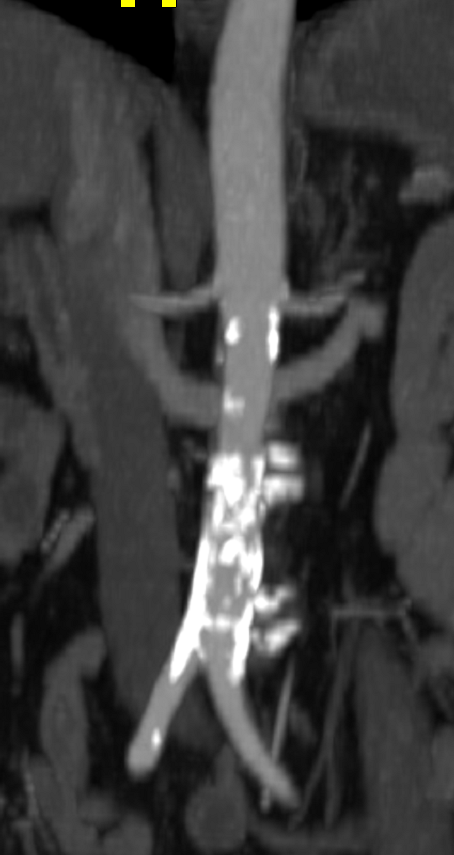

Supplement: sj-png-3-jet-10.1177_15266028241270650 – Supplemental material for Intravascular Lithotripsy and Aortic Bare-Metal Stenting: A Low-Profile Solution for the Treatment of Heavily Calcified Aorto-Iliac Disease [file sj-png-3-jet-10.1177_15266028241270650.png]

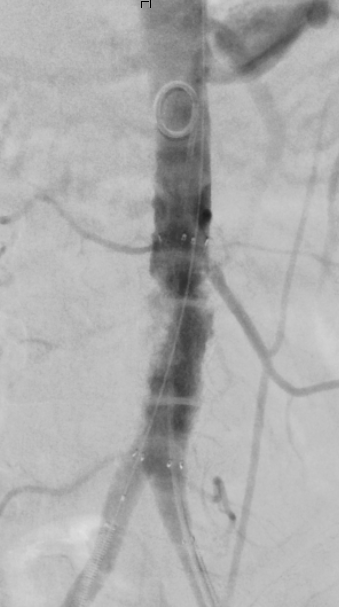

Supplement: sj-png-4-jet-10.1177_15266028241270650 – Supplemental material for Intravascular Lithotripsy and Aortic Bare-Metal Stenting: A Low-Profile Solution for the Treatment of Heavily Calcified Aorto-Iliac Disease [file sj-png-4-jet-10.1177_15266028241270650.png]

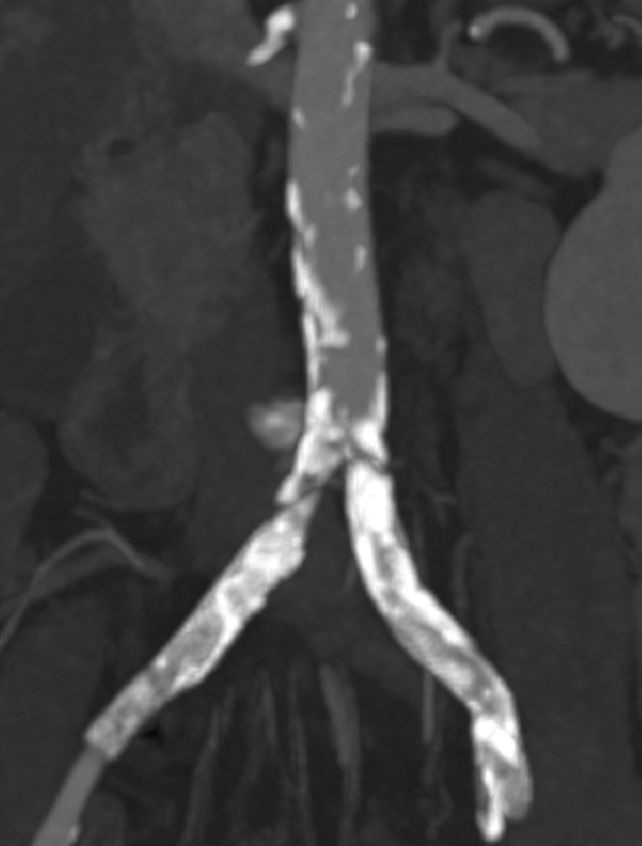

Supplement: sj-png-5-jet-10.1177_15266028241270650 – Supplemental material for Intravascular Lithotripsy and Aortic Bare-Metal Stenting: A Low-Profile Solution for the Treatment of Heavily Calcified Aorto-Iliac Disease [file sj-png-5-jet-10.1177_15266028241270650.png]

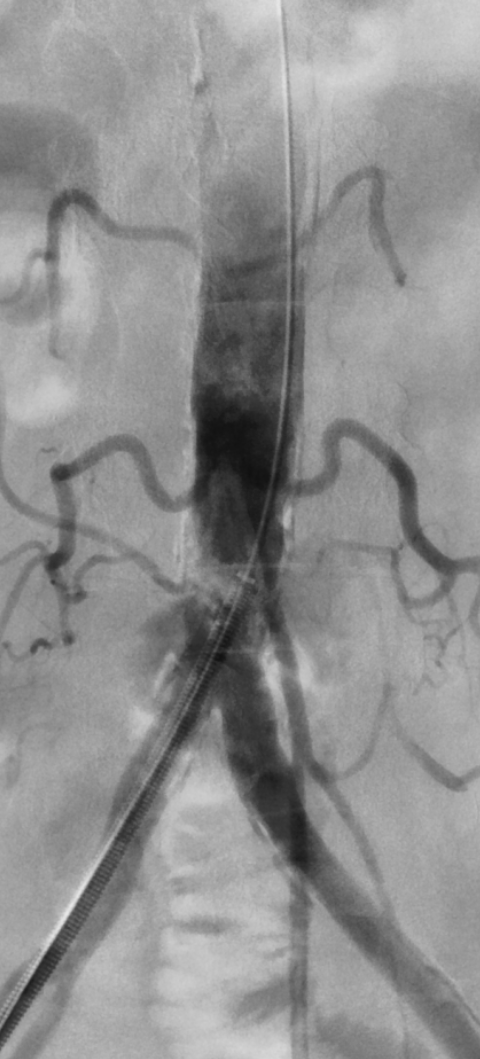

Supplement: sj-png-7-jet-10.1177_15266028241270650 – Supplemental material for Intravascular Lithotripsy and Aortic Bare-Metal Stenting: A Low-Profile Solution for the Treatment of Heavily Calcified Aorto-Iliac Disease [file sj-png-7-jet-10.1177_15266028241270650.png]

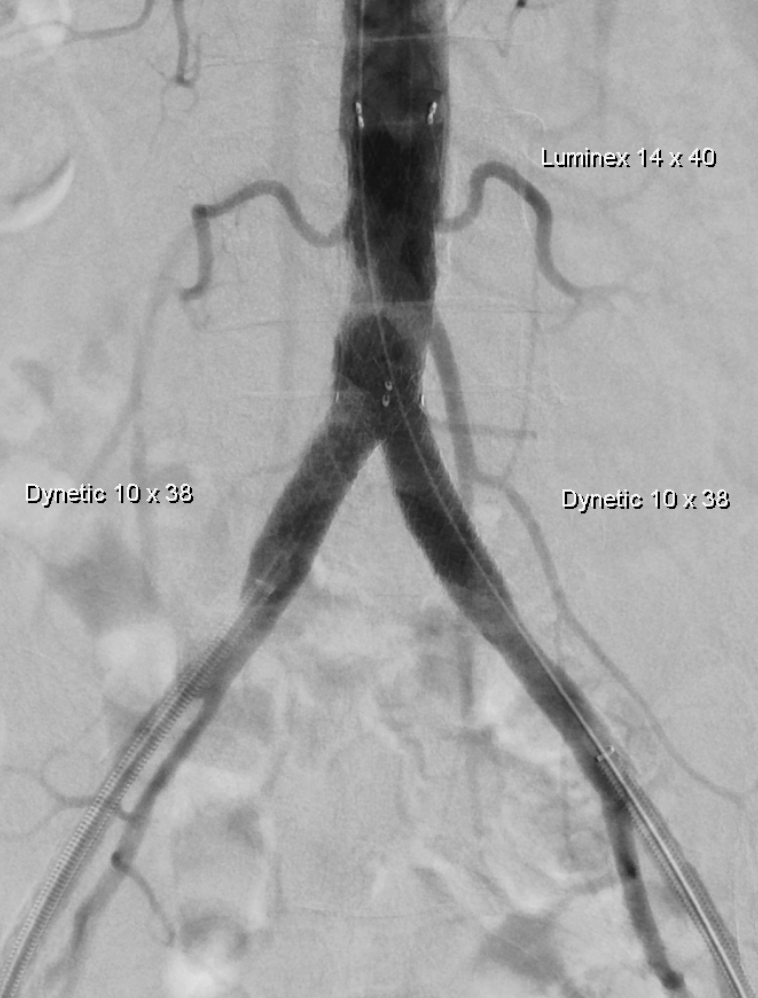

Supplement: sj-png-8-jet-10.1177_15266028241270650 – Supplemental material for Intravascular Lithotripsy and Aortic Bare-Metal Stenting: A Low-Profile Solution for the Treatment of Heavily Calcified Aorto-Iliac Disease [file sj-png-8-jet-10.1177_15266028241270650.png]
